# Streamlined and quantitative detection of chimerism using digital PCR

**DOI:** 10.1038/s41598-022-14467-5

**Published:** 2022-06-17

**Authors:** Fabian P. Suchy, Toshiya Nishimura, Shinsuke Seki, Adam C. Wilkinson, Maimi Higuchi, Ian Hsu, Jinyu Zhang, Joydeep Bhadury, Hiromitsu Nakauchi

**Affiliations:** 1 Institute for Stem Cell Biology and Regenerative Medicine, Stanford University School of Medicine, Stanford, CA 94305 USA; 2 Experimental Animal Division, Bioscience Education and Research Support Center, Akita University, Akita, 010-8543 Japan; 3 Institute of Biomedicine, Sahlgrenska University Hospital, University of Gothenburg, 41345 Gothenburg, SE Sweden; 4 Division of Stem Cell Therapy, Institute of Medical Science, University of Tokyo, Tokyo, 108-8639 Japan; 5 Department of Genetics, Stanford University, Stanford, CA 94305 USA

**Keywords:** Stem-cell biotechnology, Development

## Abstract

Animal chimeras are widely used for biomedical discoveries, from developmental biology to cancer research. However, the accurate quantitation of mixed cell types in chimeric and mosaic tissues is complicated by sample preparation bias, transgenic silencing, phenotypic similarity, and low-throughput analytical pipelines. Here, we have developed and characterized a droplet digital PCR single-nucleotide discrimination assay to detect chimerism among common albino and non-albino mouse strains. In addition, we validated that this assay is compatible with crude lysate from all solid organs, drastically streamlining sample preparation. This chimerism detection assay has many additional advantages over existing methods including its robust nature, minimal technical bias, and ability to report the total number of cells in a prepared sample. Moreover, the concepts discussed here are readily adapted to other genomic loci to accurately measure mixed cell populations in any tissue.

## Introduction

Chimeric animal models have broad and important applications in the scientific and medical community. For example, stem and progenitor cells are injected into animals to characterize engraftment and function^1^, patient-derived xenografts are used to explore tumor growth in vivo^2^, and most transgenic animals are generated via chimeric founders. More recently, systemic animal chimeras have even been created to explore developmental biology and organogenesis, with novel applications such as generating transplantable human organs^3–6^. Determining accurate levels of chimerism in these research models is necessary to assess experimental phenotypes and judge treatment efficacy. However, measuring chimerism can be challenging, as detailed below.

Flow-cytometry (FCM) is extensively used for the precise quantitation of mixed cell populations. Before analysis by FCM, tissues must be dissociated into a single-cell suspension. Fluorescently labelled antibodies or transgenically-expressed fluorophores can then be used to identify specific cell populations when run through a flow-cytometer. In the context of animal chimeras, if the cells are significantly different (e.g., human and mouse), antibodies with high specificity are available to distinguish the two populations^7^. For detecting chimerism in allogenic samples, constitutively-expressed fluorescent transgenes are frequently used for identification^6^. However, these approaches have multiple limitations. First, fluorescent transgenes often silence, and silencing can be biased by cell type^8^. Second, few antibodies are available to distinguish populations with high genetic similarities. Third, single-cell dissociation may be incomplete or result in cell death for specific tissues, thus imparting a dissociation bias. Although antibodies such as anti-mouse CD45.1 and CD45.2 have been designed to study differences in syngenic and allogenic strains^9^, phenotypic differences have been recently observed between the C57BL/6 CD45.1 and CD45.2 strains^10^. Therefore, other quantification methods are needed that are resistant to silencing, have less technical bias, and can distinguish subtle differences between two similar cell populations.

**Figure 1:**
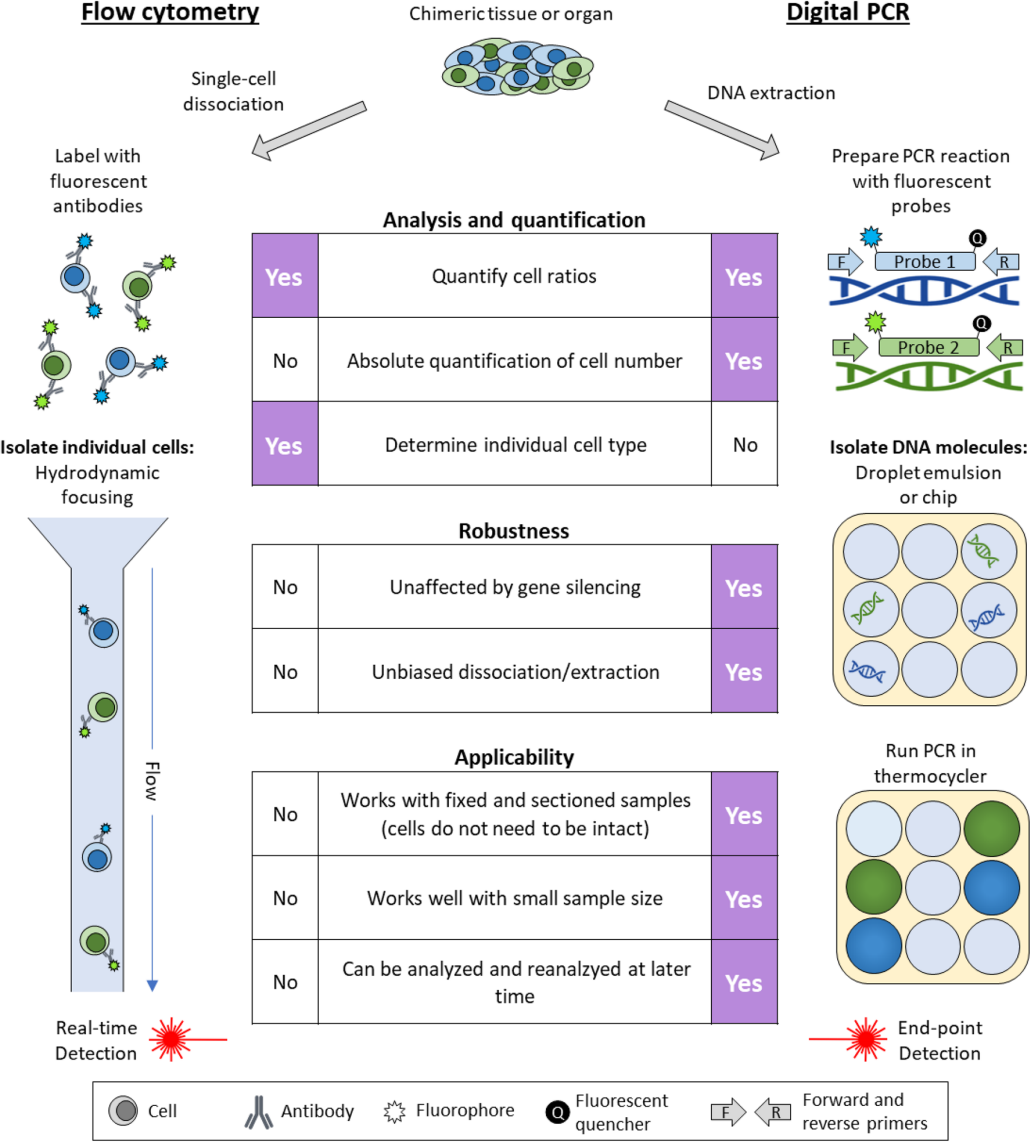
Juxtaposition of flow cytometry and digital PCR for basic chimerism analysis.

Digital PCR (dPCR) is an analytical tool that can be used as an alternative to flow-cytometry for absolute quantification of chimerism with many benefits (Fig. 1). Since all cells are lysed and DNA extracted in preparation for dPCR, tissue samples can be frozen, fixed, or even sectioned. Thus, samples can be prepared and analyzed later. In addition, cell-lysis protocols are generally more powerful and have less tissue-specific biases than enzymatic cell-dissociation buffers^11^. This eliminates dissociation bias and greatly streamlines sample preparation. Regarding specificity, reactions can be prepared with primers and hydrolysis-probes that distinguish a single nucleotide difference. This enables the flexibility to quantify ratios of nearly identical cell types.

The dPCR detection strategy is more robust and quantitative than other PCR-based approaches. After sample preparation, each dPCR reaction is partitioned into thousands of microreactions, such that some contain a single copy (or few copies) of target DNA, while many other partitions contain no target^12^. This is conceptually similar to flow-cytometry, during which cell-suspensions are hydrodynamically focused into a single column and then may be partitioned into individual droplets. After thermocycling, the dPCR microreactions are scored as positive or negative (digital output) from which the precise concentration of target DNA can be discerned. In contrast to common PCR and qPCR methods, the quantification strategy for dPCR does not require as consistent or efficient amplification. dPCR is therefore more resistant to inhibitors and results in absolute quantification without references, as opposed to the relative quantification used in qPCR^13^.

Here we describe a novel method for analyzing chimerism across common albino and non-albino mouse strains using a single-nucleotide discrimination (SND) droplet digital PCR assay (ddPCR is type of dPCR that uses microdroplets to partition the PCR reaction)^12^. The assay is extensively characterized with numerous mouse strains, dilution series, and crude lysates from various tissues. Although the assay developed here has direct utility, concepts developed and discussed in this manuscript can be adapted to quantify chimerism or mosaicism in almost any experimental setting.

## Results

### Development and optimization

**Figure 2:**
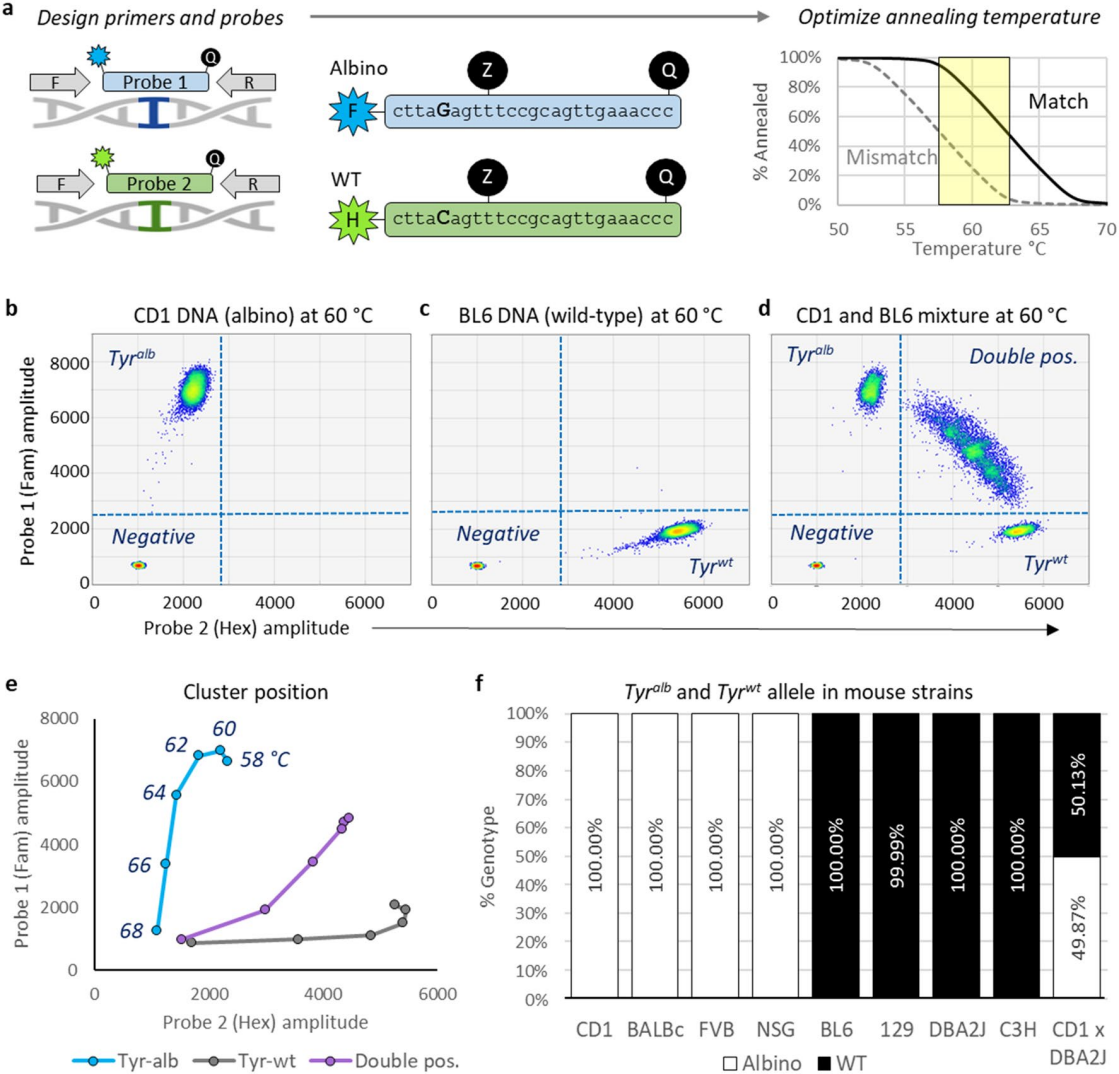
SND-ddPCR assay development and optimization. (**a**) Schematic representing primer/probe annealing and optimization strategy for the SND-ddPCR assay. Left: F and R indicate shared forward and reverse primers, respectively. Probes 1 and 2 are hydrolysis probes with FAM (blue) or HEX (green) fluorophores on the 5′ (left) end, and a quencher on the 3′ (right) end. Middle: Sequence of probes used to detect albino or WT allele. Internal ZEN quencher (Z) was used to reduce background. A bold letter indicates single-base difference between probes. Right: Theoretical melt curve for hydrolysis probes. The solid line represents probe annealing when all bases are matched to the target; the dashed line represents annealing with a single base mismatch. The ideal temperature range for SND assays is highlighted in yellow, which results in maximum separation between matched and mismatched annealing. (**b**–**d**) 2D display of SND-ddPCR results. Clusters composed of positive or negative microreactions are shown as a heat map in each quadrant. CD1 (1362 copies/µl), BL6 (1353 copies/µl) and CD1 + BL6 mixture (combined 2719 copies/µl) are shown in (**b**), (**c**), and (**d**), respectively. (**e**) 2D display showing centroid of the Tyr^Alb^, Tyr^WT^, and the double-positive cluster when analyzed at various annealing temperatures. The highest temperature is shown at the bottom left, and the lower temperatures move along the respective line upward and/or rightward. (**f**) Frequency of Tyr^Alb^ and Tyr^WT^ allele detected in various mouse strains. CD1xDBA2J is an F1 hybrid.

Many common laboratory mouse strains carry homozygous guanine to cytosine substitutions in the tyrosinase (*Tyr*) gene. This causes a p.(Cys103Ser) amino acid change, which fails to produce melanin, producing the albino phenotype^14^. We designed an SND-ddPCR assay to differentiate and quantitate the abundance of the wild-type (*Tyr*^*WT*^) and albino (*Tyr*^*alb*^) tyrosinase alleles. The assay utilized forward/reverse primers that flank Cys103 to amplify a 100 bp region, a 6-carboxyfluorescein (FAM) labeled hydrolysis probe that matches the albino allele, and a hexachloro-fluorescein (HEX) labeled hydrolysis probe that matches the wild-type allele (Fig. 2a). As these probes differ only by a single nucleotide and are required to be multiplexed in each reaction, incorrect annealing to the mismatched amplicon is inevitable. As depicted in Fig. 2a (right), optimizing the ddPCR annealing temperature results in minimal mismatch annealing and maximal match annealing.

Column-purified CD-1 (CD1, albino) and C57BL/6 (BL6, wild-type) genomic DNA were first analyzed in different reactions with an annealing temperature of 60 °C (Fig. 2b, c). The assay worked as expected: a negative cluster that contained droplets without *Tyr* was in the lower left quadrant; droplets containing *Tyr*^*Alb*^ from the CD1 mouse strain were detected with the FAM probe in the upper left quadrant (Fig. 2b); droplets containing *Tyr*^*WT*^ from the BL6 mouse strain were detected with the HEX probe in the lower right quadrant (Fig. 2c). CD1 and BL6 DNA were then mixed at a 1:1 ratio and characterized by ddPCR (Fig. 2d). This resulted in an arc-shaped cluster dispersed throughout the upper right quadrant, which contains droplets with at least one copy of both *Tyr*^*Alb*^ and *Tyr*^*WT*^. This cluster shape is typical of SND-ddPCR assays and is a result of *partition-specific competition*^15^. However, high concentrations of *Tyr* could lead to a broadening of the double-positive cluster, hindering the resolution from other clusters. Since counting the droplets in each of the four quadrants is critical to the accuracy of this assay, an upper limit of ~ 8 ng/µl mouse genomic DNA (~ 3000 copies of *Tyr*/µl) was selected for future reactions.

Figure 2b indicates that the HEX probe was mismatch-annealing (i.e., cross-reacting) with *Tyr*^*Alb*^, as indicated by the slight rightward shift (i.e., leaning) of the *Tyr*^*Alb*^ cluster when compared to the negative cluster. The reciprocal is true of the FAM probe with *Tyr*^*WT*^ (Fig. 2c). Therefore, the reactions were rerun at multiple annealing temperatures to find conditions optimal for cluster separation (Fig. 2e, Supplementary Fig. 1, Supplementary Table 1). Although there was less mismatch-annealing ≥ 64 °C, the match-annealing started to drop. Maximum cluster separation was achieved at 60–64 °C. All further reactions were run at 60 °C, and gating was performed in quadrants as shown in Supplementary Fig. 2.

### Characterization of common mouse strains

Having optimized the SND-ddPCR assay for detection of the *Tyr*^*Alb*^ and *Tyr*^*WT*^ alleles, we used the assay to genotype different mouse strains. Genomic DNA was column purified from white mice (CD1, BALB/c FVB/NJ, NSG) and black/brown mice (BL6, 129, DBA/2J, C3H). The white mice only had the albino allele and the black/brown mice primarily had the WT allele (Fig. 2f, Supplementary Table 2). To further validate our system, we generated *Tyr*^*Alb/WT*^ hybrid mice by crossing a CD1 male with a DBA/2J female. As expected, DNA analyzed from this hybrid strain showed a 1:1 ratio of *Tyr*^*Alb*^:*Tyr*^*WT*^. These data suggest that the SND-ddPCR assay could be used to detect chimerism between any of the white and brown/black mice analyzed.

### Dilution series: trueness and precision

**Figure 3:**
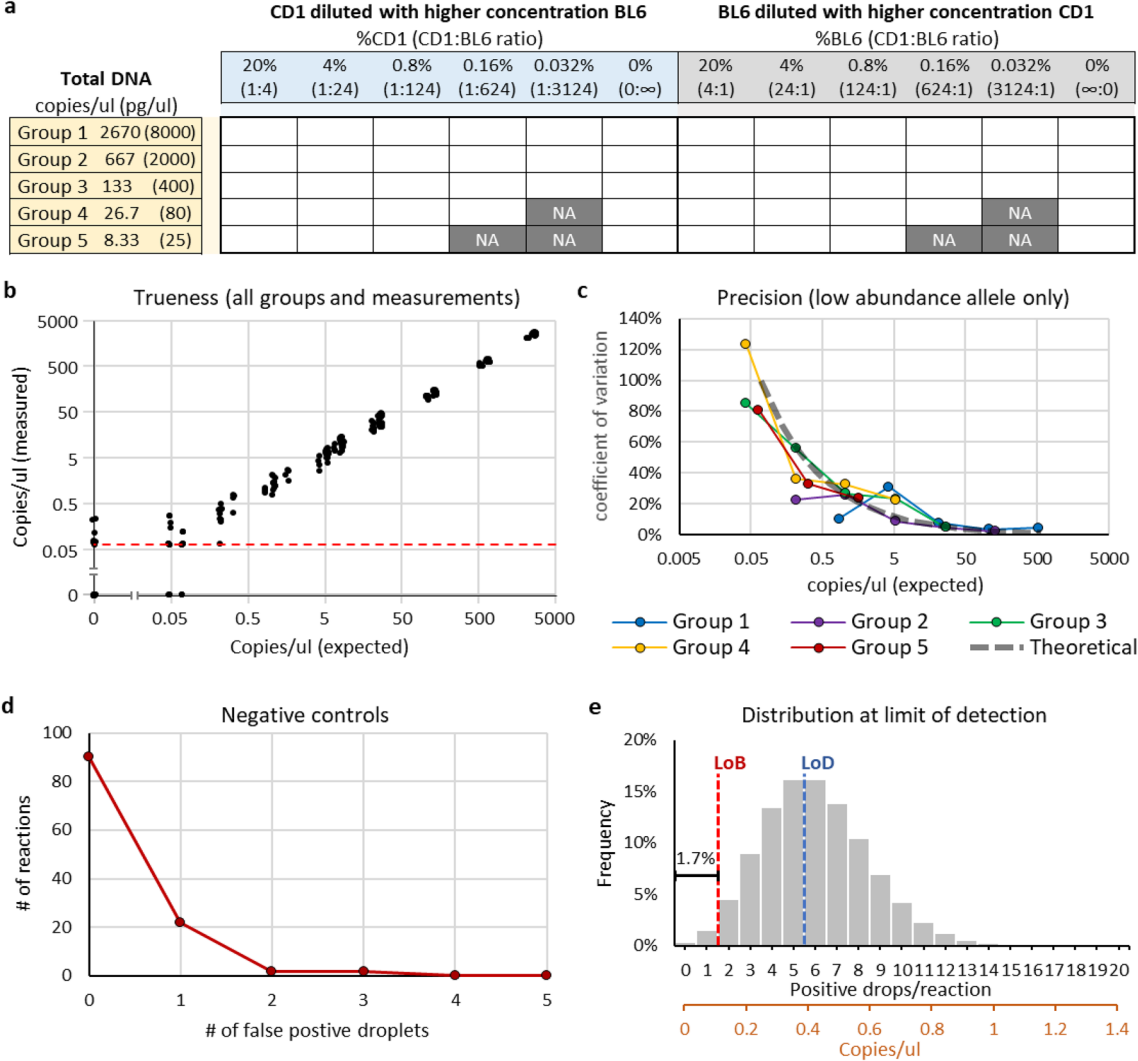
Trueness and precision at various dilutions. (**a**) Dilution table of CD1 and BL6 genomic DNA mixtures. Total DNA concentration was the same for each group. Dark gray boxes labeled NA were not analyzed because the concentration of low-abundance DNA was less than 0.5 molecules per reaction. (**b**) Combined Groups 1–5 concentration measurements compared to expected concentration; axes are log-scaled. 5% random error added to each point to aid in distinguishing overlapped datapoints. The red dashed line indicates the concentration at which there is only a single positive droplet per reaction (single molecule detection limit). (**c**) Coeffecient of variation (CoV, standard deviation divided by mean) calculated for the low-abundance allele in Groups 1–5 (n = 4 for each point: combined low-abundance CD1 and BL6 measurements, and two replicates of each). Gray dashed bar approximates theoretical CoV from subsampling error. (**d**) The number of false positive droplets in negative controls (n = 116). (**e**) Theoretical Poisson distribution of measured concentration when a sample is prepared at the LoD (6 molecules per reaction). The red dashed line and the blue dashed line indicate LoB and LoD, respectively. Black x-axis shows concentration as positive drops/reaction; brown x-axis shows concentration as copies/µl (assume 17,000 total drops/reaction). Area to the left of the LoB is β error (1.7%).

To validate the trueness and precision of the SND-ddPCR assay at various allele ratios and concentrations, a dilution matrix was prepared (Fig. 3a, Supplementary Table 3). The chimerism varied from 20 to 0.03%, and the total concentration ranged from 2670 to 8.3 copies/µl (8 ng/µl to 25 pg/µl, respectively). The assay could accurately report concentration and %chimerism at all concentrations above 0.5 copies/µl (Fig. 3b; Supplementary Fig. 3). Detection was linear across a broad range (Supplementary Fig. 4). Importantly, the concentration of the high abundance allele did not noticeably influence the detection of the low abundance allele. Precision also decreased significantly below 0.5 copies/µl as seen by a marked increase in variation (Fig. 3c), however precise detection of the low abundance allele was not influenced by the quantity of the high abundance allele. The measured variation resembled the theoretical variation in ddPCR at low concentrations, which is primarily due to random sampling distribution (R^2^ = 0.79, Fig. 3c). Collectively, three important conclusions can be drawn: (1) detection of the low abundance allele is not influenced by the high abundance allele, thus loading more DNA will increase the sensitivity; (2) trueness and precision are not notably skewed from theoretical expectations; and (3) the assay works well for reciprocal detection of either rare *Tyr*^*Alb*^ or rare *Tyr*^*WT*^.

### LoB and LoD

Understanding the limits and confidence intervals of analytical tools is critical to interpreting results. Therefore, we next established a limit of blank (LoB) and a lower limit of detection (LoD). The false-positive rate was considered to set an appropriate LoB, such that at the set LoB there would be a < 5% chance of α error (false positives). Although the theoretical detection limit of PCR is a single molecule, up to three false positive droplets were detected in the reactions analyzed in Fig. 3a (Supplementary Table 3). A total of 12 drops were positive in the 20 negative controls, giving an average false-positive rate (A_FP_) of 0.6. Notably, A_FP_ was higher for *Tyr*^*Alb*^ (0.9) than *Tyr*^*WT*^ (0.3). Also, the A_FP_ was higher in groups with more total DNA (1.75, 0.5, 0.25, 0.25 and 0.25 for Groups 1–5, respectively). Since DNA was extracted from both BL6 and CD1 simultaneously, it is possible the false positives were due to cross-contamination. Therefore, an additional six DNA extractions were performed (three for BL6 and three for CD1) on separate days. Each extraction was analyzed by the SND-ddPCR assay 16 times at the higher concentration of 8 ng/µl, totaling 96 additional negative controls (Supplementary Table 4). Indeed, A_FP_ was lower with no notable difference between *Tyr*^*Alb*^ (0.167) and *Tyr*^*WT*^ (0.25). All 96 + 20 negative controls were combined, giving a final A_FP_ of 0.224 (Fig. 3d). The LoB was set to 2 positive drops/reaction which would give a false positive rate of 3.4% (4 reactions out of 116) in this data set.

The LoD was established such that there would be a < 5% chance of β error (false negatives). This was achieved at 6 droplets/reaction (~ 0.42 copies/µl), for which there was only a 1.7% chance that a sample would fall below the LoB and not be detected (Fig. 3e). Given an A_FP_ of 0.224, our values for the LoB and LoD were checked using published guidelines and the results were identical^16^.

### Blood chimerism: ddPCR vs flow cytometry

**Figure 4:**
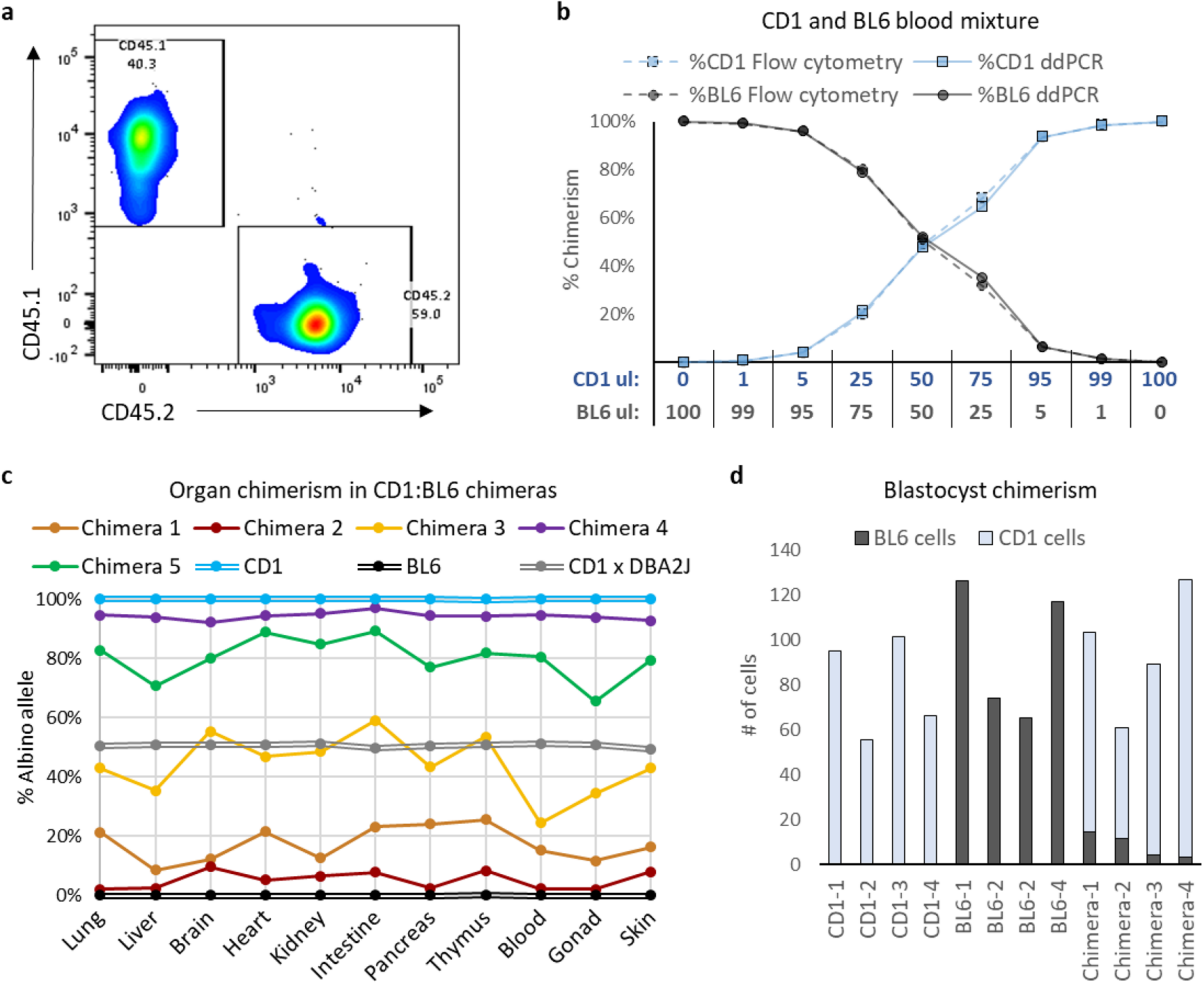
Validation in various organs and tissues. (**a**) Flow cytometry plot of a CD1 and BL6 mouse peripheral blood mixture stained with CD45.1-PE-Cy7 (y-axis, CD1 strain) and CD45.2-BV421 (x-axis, BL6 strain) antibodies. (**b**) Bottom: CD1 and BL6 blood mixed at different volumetric ratios. Top: Measured chimerism of CD1 and BL6 blood mixtures. Measured by FCM using CD45.1/CD45.2 antibodies (dashed lines) or SND-ddPCR assay (solid lines). (**c**) Chimerism was measured with crude lysates of various organs from 5 chimeric mice (CD1:BL6 chimeras). CD1, BL6 and CD1xDBA2J F1 hybrid are non-chimeric controls. (**d**) Number of BL6 and CD1 cells in blastocysts (1 lysed blastocyst per reaction). 4 CD1, 4 BL6 and 4 chimeric blastocysts (CD1 injected with BL6 embryonic stem cells) were analyzed.

Up to this point, all analyses were performed with column-purified samples of genomic DNA that had been mixed to simulate chimeric samples. It is important to determine if the SND-ddPCR assay can accurately report chimerism when DNA is extracted from actual chimeric tissues. Blood is a convenient organ with which to validate this assay because it can be mixed at various ratios, readily analyzed by FCM, and directly compared to ddPCR without concern of heterogeneity or cell-dissociation bias. Since CD1 and BL6 mice express CD45.1 and CD45.2, respectively, fluorescently labelled antibodies can distinguish the two populations (Fig. 4a). Blood from CD1 and BL6 mice were mixed at various ratios (Fig. 4b bottom, Supplementary Table 5) and further split into two aliquots for DNA extraction or antibody staining. Chimerism was analyzed from the extracted DNA by ddPCR and compared to chimerism measured by FCM (Fig. 4b top). The measurements from both samples were nearly identical, with an R^2^ value of 0.998. This validates the assay for accurate detection of blood chimerism.

### Organ/tissue chimerism: analysis with direct lysis buffer

Purification of DNA from many samples can be tedious. Analysis of DNA directly from lysed cells without further purification would streamline the SND-ddPCR assay’s pipeline. To determine if crude cell-lysate from organs can be directly run on ddPCR and analyzed for chimerism, individual organs were harvested and digested with a tissue lysis buffer. The organs were harvested from CD1:BL6 chimeras (n = 5) and three different controls, and the crude-lysate loaded directly into the ddPCR reaction. As expected, the degree of overall chimerism varied significantly from mouse to mouse, ranging from 6 to 96%; however, chimerism in the organs within the same mouse was relatively stable (Fig. 4c, Supplementary Table 6). The CD1 and BL6 controls were only positive for their respective alleles. A CD1 x DBA/2J F1 hybrid was also correctly 50% *Tyr*^*Alb*^ and 50% *Tyr*^*WT*^ in all organs. We then analyzed F1 hybrid DNA after column purification and found no significant difference (*p* = 0.43) compared to crude lysate analyses (Supplementary Fig. 5a). However, the average yield of DNA was 5.8-fold higher when using crude lysate (Supplementary Fig. 5b). These data suggest that SND-ddPCR assays can be used to accurately measure low to high chimerism in all organs, without the need for extracting clean nucleic acid.

One of the earliest and smallest structures during development is the blastocyst. To determine if the SND-ddPCR assay could detect chimerism in these small structures, individual blastocysts were lysed and analyzed as a single blastocyst per reaction. Four CD1 and four BL6 control embryos were positive for only the *Tyr*^*Alb*^ or *Tyr*^*WT*^ allele, respectively (Fig. 4d, Supplementary Table 7). In contrast, four CD1 embryos injected with a few BL6 mouse embryonic stem cells (mESCs) were all positive for both *Tyr*^*Alb*^ and *Tyr*^*WT*^ (Chimera-4 was below the LoD). Additionally, since the entire blastocyst was loaded in one reaction, theoretically, we could calculate the total number of cells. An average of 90 cells/blastocyst were detected, which is in agreement with the expected number of cells in a late blastocyst. ddPCR’s ability to count cells within a blastocyst was further explored by staining the DNA of an additional 15 blastocysts with the fluorescent dye Hoescht. Each embryo was individually imaged and analyzed on ddPCR. Although counting nuclei proved challenging, the overall intensity of Hoescht strongly correlated with the with numbers of cells counted by ddPCR (Supplementary Fig. 6a,b). In addition, we diluted hemolysed mouse blood to 4 different concentrations and compared the cell count using ddPCR to an automated cell-counter. The counts were similar (Supplementary Fig. 6c), demonstrating that ddPCR can accurately count cells at various concentrations. Therefore, the SND-ddPCR assay can be used to determine chimerism and overall numbers of cells in small structures that could not be readily analyzed by FCM, weight, and other methods.

## Discussion

Robust quantification of chimerism is critical to measuring experimental outcomes. Here, we extensively validate a novel chimerism detection method that can be immediately implemented for many mouse strains and experimental workflows. We also showcase optimization and validation strategies that can be readily adapted to other assays. Since the method is a genetic assay, it has many attractive qualities, including (1) the ability to distinguish a single nucleotide difference among otherwise identical cells, (2) the fact that transgenic modifications are not required, (3) agnosticism to gene silencing, (4) resistance to tissue/cell-type dissociation bias, and (5) streamlined workflow.

One of the most powerful and unique capabilities of dPCR is the ability to robustly quantify allelic ratios that differ by only a single nucleotide. Most cells acquire point mutations during division, thus even subclones from the same parent line can be distinguished. Although we used an SND assay to identify a common SNP among different mouse strains, SND assays can be applied to most SNPs, including mosaic and cancerous genotypes, as previously shown in human patients^17,18^. The ddPCR-based SND assay differs from other SND assays that rely on mismatches in the primer at the 3′-end. First, primer-mismatched assays require two separate reactions. Second, the polymerase will eventually extend the mismatched primer, even if the mismatch is in the 3′ end^19^. Once this occurs, the PCR will continue with normal efficiency and a false-positive signal will be detected. Finally, the ultra-quantitative nature of ddPCR not only reports the presence or absence of a SNP, but also reliably reports the concentration of each allele nearly independent of overall concentration.

The use of a single, PCR-compatible lysis buffer can streamline the detection of hundreds of samples. This can be performed in a high-throughput format (e.g., 96-well plate) with less hands-on time and minimal attention to individual samples. In contrast, cell dissociation for FCM requires tissue-specific and optimized lysis conditions, individual focus on each sample, and multiple spin/wash steps^11^. Additionally, most FCM samples need to be analyzed the same day before the cells die. Because of the easier and more reliable workflow, we have transitioned many of our chimerism analyses across organs from FCM to ddPCR^20^.

The dPCR detection strategy is the most robust and quantitative method compared to other PCR-based approaches. This is because the quantification strategy for dPCR does not require consistent or efficient amplification, but instead relies on simply counting the number of positive microreactions in individual clusters. Consequently, dPCR is more resistant to inhibitors and results in absolute quantification without references, as opposed to the relative quantification used in qPCR. However, even dPCR can be overwhelmed with inhibitors, preventing amplification^13^. Although we could directly analyze crude lysates from all tested solid organs and tissues with the SND-ddPCR assay, it did not work well for whole blood. This was easily remedied with a hemolysis step, which is usually performed before FCM analysis as well. An alternative is to analyze a smaller volume of blood, but this results in a loss of sensitivity.

When using a genetic assay to determine chimerism, ploidy must be considered. Liver, placenta, and cancer cells are often polyploid, which could lead to an overestimation of cell number^21,22^. Similarly, rapidly dividing cells spend a large proportion of time in the S and G2 phases of the cell cycle, during which they have additional copies of chromosomes. In contrast, mature gametes are haploid, and red blood cells (RBCs) lack nuclei, minimizing or precluding their detection, respectively. However, except for RBCs, most changes in ploidy will only lead to a calculation error within a factor of 2, which is similar to the uncertainty associated with qPCR^23^. In addition, the lack of nuclei in RBCs is mostly advantageous because it diminishes a blood measurement bias that would otherwise occur in heavily vascularized organs.

Perhaps the most frustrating property of ultra-sensitive assays is the potential for false positives^24–26^. Since PCR can detect a single molecule, trace amounts of carry-over from other reactions can lead to false positives. Initially, 8 of 20 negative control reactions had at least one false-positive drop. In our lab, we occasionally PCR amplify and sequence the tyrosinase allele. Therefore, the false positive droplets in the SND assay could be due to post-PCR contamination from other experiments^27^. Alternatively, simultaneous handling of samples that contain high concentrations of *Tyr*^*Alb*^ and *Tyr*^*WT*^ could easily result in cross-contamination. Extra precautions (e.g., glove changes, wrapping tubes, additional washes) were utilized in repeated experiments to avoid contamination, and the false positive rate decreased. Each lab is recommended to determine its own false positive rate and develop controls to prevent cross-experiment contamination.

It is important to understand the limits and confidence intervals of analytical platforms. Our LoB and LoD were established to have < 5% α error and < 5% β error, respectively. This means that a sample at the LoD will be detected > 95% of the time if using the LoB as a cutoff. However, confidence intervals determined from theoretical models (e.g., Poisson distributions) do not account for experimental, biological, or equipment variability, which will likely increase uncertainty^28^. Thus, caution is appropriate. Numerous controls should be analyzed (and reanalyzed) and more stringent cut-offs should be selected if needed. It is also important to be careful when setting detection limits in terms of copies/µl or cells/reaction because these terms are dependent on the volume of sample analyzed. Some of our calculations assume there are 17,000 partitions (close to our average number of droplets per reaction), with a droplet volume of 0.85 nl (determined by BioRad). This corresponds to a total volume of 14.5 µl analyzed per reaction. Although at least 20 µl is usually prepared for each reaction, approximately 25% is lost during the partitioning process. Therefore, if the droplet volume or number of droplets decreases, the precision and sensitivity also decrease, and confidence intervals should be reassessed. This is most important near the lower limits of detection and becomes negligible as concentration increases.

The SND-ddPCR assay accurately measured chimerism in all organs that were homogenized with lysis buffer and analyzed using the crude lysate. The hybrid control, which had an exact 1:1 ratio of *Tyr*^*Alb*^ and *Tyr*^*WT*^ in all cells, accurately reported this ratio in the tested organs. This shows that no allele bias occurs during processing. The diverse range of average chimerism among the different chimeras highlights the variability of these types of microinjection experiments, emphasizing the need for robust quantification to measure subtle changes. In contrast, the chimerism among various organs in a single mouse was quite similar. This is expected because mESC engraftment occurs at an early stage, and the mESCs we injected should not have a developmental predisposition to any tissues. Thus, in these experiments, chimerism is established stochastically at early timepoints, and relatively evenly distributes to all tissues. Similar experiments could be performed to discover tissue distribution biases of different cell lines.

We demonstrated the unique ability of the SND-ddPCR assay to count the overall number of cells in a sample by lysing and directly analyzing entire mouse blastocysts. With small samples, it is often impossible to dissociate into single cells without significant sample loss. Thus, this assay can be used as a primary or supplemental approach to measure organ/tissue size in small chimeric samples.

Reliable and robust measurements are key to scientific reproducibility. By simplifying data acquisition, researchers will generate higher resolution datasets with greater fidelity. dPCR chimerism assays are relatively easy to develop and extremely flexible. In addition, they have similar single-cell limitations compared to FCM-based assays, with numerous advantages. Although other technologies, such as NGS, are being adapted to analyze human blood chimerism^29,30^, the time/cost burden is still currently too high for rapid, low-cost, and flexible measurements. Therefore, we believe dPCR is a critical tool that should continue to be used to streamline and strengthen chimerism/mosaicism measurements with high accuracy and reliability.

## Materials and methods

### Mice

All animal experiments were approved by the Administrative Panel on Laboratory Animal Care at Stanford University (APLAC #29042), and all experiments were performed within relevant guidelines and regulations. Mice were purchased from The Jackson Laboratory or Charles River and housed with free access to food and water. Euthanization was performed by introducing carbon dioxide into the animal cage at a flow rate of 30% cage volume per minute and followed by cervical dislocation. Animal data is reported in accordance with ARRIVE guidelines.

### Column purified DNA

Genomic DNA was purified using the DNeasy Blood and Tissue Kit (QIAGEN, Hilden, Germany) following the recommended protocol. For SND-ddPCR assay optimization (CD1 and BL6) and mouse strain genotyping (CD1, BALBc, FVB, NSG, BL6, DBA2J, CD1xDBA2J), DNA was extracted from 1 to 2 mouse ear punches (~ 2 mm in diameter). Tissue lysis and homogenization were achieved after 3 h at 55 °C followed by pipet trituration. For genotyping the 129 and C3H strains, DNA was extracted from 0.1 to 1 million cultured cells. For blood, 30–50 µl was lysed and purified per Qiagen’s protocol. For organs, a cube of tissue (~ 1.5 mm^3^) was dissected from the organs of a euthanized mouse. The tissue was disrupted in Qiagen’s tissue lysis buffer by heating ~ 12 h at 55 °C and homogenizing with a pipet. All samples were eluted in 100–200 µl of elution buffer AE. Specific sample detail regarding yields and volume are shown in Supplementary Tables 1–7.

### SND-ddPCR assay

Under the 2020 guidelines regarding the minimum information necessary for the publication of quantitative digital PCR experiments, a dMIQE2020 table was added as Supplementary Table 8^31^.

Primers were designed to amplify a 100 bp region of the mouse tyrosinase gene (NCBI ID 22173, Mus musculus chromosome 7), which differs by a single nucleotide between albino and non-albino mice. Primers were validated for specificity in silico with NCBI’s primer designing tool Primer-BLAST (https://www.ncbi.nlm.nih.gov/tools/primer-blast/index.cgi). Two hydrolysis probes with different fluorophores were used to detect either the albino allele or the wild-type allele in a single reaction with the above primers. Primers and probes were from IDT (Coralville, IA); probes contained the fluorophore at the 5′ end, the IowaBlack quencher at the 3′ end, and an additional internal ZEN quencher. All oligos were stored in the dark at 4 °C for less than 1 year in TE buffer. Primer/probe type, name, reaction concentration, and sequence are listed below:

- Forward; mTyr-F/1; 1.8 µM; AATAGGACCTGCCAGTGCTC
- Reverse; mTyr-R/1; 1.8 µM; TCAAGACTCGCTTCTCTGTACA
- Albino probe; mTyr-alb-P/1; 0.25 µM; FAM-cttaGagtttccgcagttgaaaccc-Zen/IowaBlack
- Wildtype probe; mTyr-wt-P/1; 0.25 µM; HEX-cttaCagtttccgcagttgaaaccc-Zen/IowaBlack.

Each ddPCR reaction was prepared and analyzed with the QX200 ddPCR system (BioRad, Hercules, CA) per BioRad’s standard recommendations for use with their ddPCR™ Supermix for Probes (No dUTP) unless otherwise stated. All reactions were mixed to 25 µl and contained up to 10 µl of DNA prepared from column purified extracts or crude lysate. For droplet generation, 20 µl were loaded into the droplet generator cassette in groups of eight per BioRad’s protocol (this requires ~ 2 min for every eight samples; ~ 25 min for 96). Thermocycler conditions: 95 °C × 10 min; 50 cycles of 94 °C × 30 s and 60 °C × 60 s; 98 °C × 10 min; hold at 4 °C. For temperature optimization experiments, the annealing/extension temperature of 60 °C was varied between 58 and 68 °C. The average number of partitions after droplet generation was 17,000. Data analysis was performed with QuantaSoft Analysis Pro version 1.0.596 (BioRad, Hercules, CA).

### Gating strategy

To maintain simplicity, gating of ddPCR cluster data was performed using the quadrant tool in QuantaSoft Analysis Pro version 1.0.596 (BioRad, Hercules, CA). As a guide, we set the gates to six standard deviations from the cluster centroids as shown in Supplementary Fig. 2b (centroids measured in QuantaSoft). Standard deviations were estimated by first measuring a peak’s full width at half the max height (FWHM) using Microsoft PowerPoint. FWHM is equal to 2.354 standard deviations, thus 2.55 × FWHM is equivalent to 6 standard deviations. Using this approach, the quadrant centers were set near coordinates (2900, 2580).

### Peripheral blood FCM analysis

Peripheral blood from up to six CD1 mice (8–12 weeks of age) was collected and pooled until reaching ~ 500 µl. The same was done with BL6 mice of similar age. The CD1 and BL6 blood samples were then mixed at various volumetric ratios indicated in Fig. 4b (final volume 100 µl for all samples). Following red blood cell lysis (hemolysis), samples were split for ddPCR analysis (column purification) and flow cytometric analysis as described previously^32^. For flow cytometric analysis, samples were stained with CD45.1-PE/Cy7 (clone A20; BioLegend, San Diego, CA) and CD45.2-BV421 (clone 104; Biolegend, San Diego, CA), washed and then run on a BD FACSAriaII, with data analysis performed using FlowJo software.

### Embryo culture and manipulation

Wild-type mouse embryos were collected according to published protocols^33^. In brief, superovulated CD1 mice less than 10 weeks old were euthanized, and 2-cell zygotes were obtained by oviduct perfusion. The zygotes were cultured in KSOM-AA medium (CytoSpring, Mountain View, CA; K0101) for 2 days and blastocyst-stage embryos were collected for cell injection. For micromanipulation, mESCs were trypsinized and suspended in ESC culture medium. A piezo-driven micromanipulator (Prime Tech, Tsuchiura, Japan) was used to drill the zona pellucida and trophectoderm under microscopy and 5–8 ESCs were introduced into blastocyst cavities near the inner cell mass. After blastocyst injection, embryos were cultured for 1–2 h. Mouse blastocysts were then transferred into uteri of pseudopregnant recipient CD1 female mice (2.5 days post coitum) to generate chimeric animals. In Fig. 4c, a total of ~ 40 blastocysts were injected and split between two pseudopregnant mice. ~ 20 healthy pups were born, from which five with skin chimerism visually ranging from low to high were used for analysis.

### ESC derivation and culture

Mouse ESC lines were derived and maintained in serum-free ES media as previously described^34^. In brief, blastocyst stage embryos were expanded for 7–10 days on mitotically-inactivated mouse embryonic fibroblasts (MEFs) in ESC culture media containing 20 ng/ml human LIF (Peprotech, Cranbury, NJ; 300-05), 1 uM PD0325901 (Tocris, Minneapolis, MN; 4192-10) and 3 µM CHIR99021 (Tocris, Minneapolis, MN; 4423/10). Outgrowths were picked and dissociated before replating in the same conditions. The ESCs were passaged every 3–4 days on MEFs in the same serum-free ES media.

### Crude extraction of DNA from organs

To lyse and homogenize solid organs, chimeric and control mice were euthanized (less than 16 weeks old) and a ~ 1.5 mm^3^ cube of the respective organ was added to 100–200 µl of direct lysis buffer. The lysis buffer contained 0.1% SDS, 5 mM EDTA, 1–2 mg/ml proteinase K (Thermo Fisher Scientific, Waltham, MA), 15 mM tris (pH 7.8), and 200 mM NaCl. Lysis was performed for 6–12 h at 55 °C. The lysate was then homogenized with a pipet or 20-gauge hypodermic needle if needed (most did not), and all samples were heated to 80 °C for 10 min. For blood, 10–50 µl was collected and hemolysis was performed before the addition of lysis buffer. Lysis was then performed for only 20 min at 55 °C, followed by 10 min at 80 °C. For all samples, debris was pelleted from the lysate by centrifugation at 6000*g*’s × 3 min, and 0.25–4 µl of the supernatant analyzed by SND-ddPCR. Specific sample details regarding yields and volume are shown in Supplementary Tables 1–7.

### Blastocyst ddPCR

CD1 and BL6 morulae were harvested as described above. Four CD1 morulae were injected with eight BL6 mESCs. The embryos were cultured until E4.5, after which they were transferred into 4 µl of lysis buffer in PCR tubes (~ 1 µl of embryo media was added during the transfer). The lysis buffer was similar to the buffer used for organs, except it contained only 0.2 mg/ml proteinase K. 5 µl of water was then added to each PCR tube bringing the final volume to 10 µl, and the tubes were heated to 55 °C for 10 min and 80 °C for 10 min. The ddPCR reaction mixture was prepared directly in the tubes containing lysed blastocysts. Later, an additional fifteen CD1 E4.5 blastocysts were imaged and then analyzed by ddPCR as described above.

### Blastocyst imaging

CD1 embryos were cultured until E4.5 from which 15 were selected with various sizes. To fluorescently label the DNA, the blastocysts were first incubated in KSOM-AA with 20 µM Hoescht 33342 (Thermo Fisher Scientific, Waltham, MA; 62249) at 37 °C for 30 min, and then KSOM-AA with 2 µM Hoescht 33342 at 37 °C for 30 min. The embryos were then imaged using the Operetta High Content Imaging System (Perkin Elmer, Waltham, MA) with the following parameters: 10 × objective; Hoescht excitation 50% and exposure 20 ms; bright field emission 10% and exposure 20 ms; focal plane in the center of the embryo. Using ImageJ 1.52p (NIH, USA), the images were cropped to 300 × 300 pixels. The background-subtracted Hoescht intensity was calculated as the average intensity of all pixels minus the median intensity of all pixels (plotted on the y-axis of Supplementary Fig. 6b). Each embryo was then analyzed by ddPCR and the cell count (calculations shown below) was plotted on the x-axis. Three empty wells were also imaged and analyzed as negative controls, all of which reported intensity and cell counts near 0.

### Cell count with ddPCR

Two 10 µl aliquots of hemolysed CD1 mouse blood were pelleted (500 g × 5 min). The first pellet was resuspended in 100 µl PBS. 1, 2, 3, and 4 µl of this sample were further aliquoted into four separate tubes containing 1.5 µl of cell-counting reagent Solution 13 (ChemoMetec, Bohemia, NY). PBS was added to each tube to bring the final volume up to 25 µl. After mixing, 10 µl of each sample was loaded into an NC-8 cell counting slide and counted with NucleoCounter NC-3000 (ChemoMetec, Bohemia, NY). The second blood pellet was lysed with 100 µl of direct lysis buffer and processed as described in “Crude extraction of DNA from organs”. 1, 2, 3, and 4 µl of this sample were further aliquoted into four separate tubes. ddPCR reactions were prepared directly in these tubes, all with a final volume of 25 µl. Cells/reaction was calculated as described in “Common calculations”; conversion to cells/ml was achieved by dividing by 0.025.

### LoB and LoD

The average false positive rate (A_FP_) was calculated from 58 samples containing just BL6 DNA and another 58 samples containing just CD1 DNA, (Supplementary Tables 3 and 4). There were a total of 24 false-positive drops in 116 reactions giving an A_FP_ of 0.224. 96.6% of the reactions contained < 2 false positive drops, while 77.6% of the reactions contained < 1 false positive drop. The LoB was set at 2 to maintain < 5% α error. The LoD was selected as 6 positives droplets/reaction, as this is the lowest number at which β error is < 5%. In other words, if a reaction is prepared at the LoD, a Poisson distribution predicts that there is < 5% chance that the reaction will be measured below the LoB. These values for LoB and LoD were further validated per published guidelines^16^. The Microsoft Excel equations used to validate the LoB and LoD were:

$$LOB = ROUNDUP(A_{FP} + (1.645 \times (A_{FP}^{0.5} )) + 0.8,0),$$

$$LOD = ROUNDUP(((1.645 + (((1.645^{2} ) + (4 \times LOB))^{0.5} ))^{2} )/4,0).$$

The equations returned values of 2 and 6 for LoB and LoD, respectively, matching our calculated values.

### Statistics and graphs

ddPCR 2D plots and histograms were generated with QuantaSoft Analysis Pro version 1.0.596 (BioRad, Hercules, CA). All other graphs were generated in Microsoft Excel and PowerPoint.

For Fig. 3b and Supplementary Fig. 4, all data points were from the dilution matrix shown in Fig. 3a (n = 54 × 2; each point was analyzed in duplicate). The average number of drops per reaction was 17,427, thus a reaction with a single positive drop would be measured around 0.068 copies/µl. 0.005 copies/µl was added to the x and y values of each datapoint to enable log transformation of 0 values. 5% random error was added to the x and y value of each datapoint to enable easier visualization of overlapped points. For Supplementary Fig. 4, all values were then log_10_ transformed, and log_10_(0.005) was subtracted from each value to shift (0, 0) to the graph’s origin. Regressions were performed with Microsoft Excel.

The CoV for each datapoint in Fig. 3c was calculated as the standard deviation divided by the mean of 4 datapoints (CD1 low abundance allele and BL6 low abundance allele in each group at matching dilutions were pooled). The theoretical CoV was estimated as the square root of the number of positive drops divided by the number of positive drops—note this estimation is less true at lower concentrations^16^. Conversion between positive drops/reaction and copies/µl was performed assuming 17,000 droplets total. The coefficient of determination (R^2^) was determined in Microsoft Excel by comparing the calculated CoV to the theoretical CoV at the measured concentrations.

Figure 3e was generated by graphing a Poisson distribution in Microsoft Excel using the following function for which *k* ranged from 0 to 20: *Frequency* = POISSON.DIST(*k*,6,FALSE).

For Fig. 4b, the coefficient of determination was determined in Microsoft Excel by comparing the %CD1 measured by ddPCR to the %CD1 measured by FCM.

A two-tailed t-test was performed in Supplementary Fig. 5 to compare chimerism measurements from two different extraction techniques. The Microsoft Excel function was *p-value* = T.TEST(*array1,array2,*2,3). An alpha value of 0.05 was used to determine significance.

### Common calculations

%Chimerism:

$$\% {\text{albino}}\,{\text{chimerism }} = \, \left[ {Tyr^{Alb} } \right]/\left( {\left[ {Tyr^{Alb} } \right] + \, \left[ {Tyr^{WT} } \right]} \right) \, \times { 1}00,$$

$$\% {\text{WT}}\,{\text{chimerism }} = \, \left[ {Tyr^{WT} } \right]/\left( {\left[ {Tyr^{Alb} } \right] + \, \left[ {Tyr^{WT} } \right]} \right) \, \times { 1}00.$$

Fractional abundance (λ, average number of copies per drop):

$$\lambda \, = {\text{ ln}}\left( {{\text{n}}_{{{\text{total}}\,{\text{drops}}}} } \right) \, {-}{\text{ ln}}\left( {{\text{n}}_{{{\text{negative}}\,{\text{drops}}}} } \right).$$

Concentration measured in each ddPCR reaction expressed in copies/µl:

$${\text{C}}_{{{\text{copies}}/\upmu {\text{l}}}} = \, \lambda /0.000{85}{\text{.}}$$

Total copies in each reaction prior to droplet generation:

$${\text{C}}_{{{\text{copies}}/{\text{reaction}}}} = {\text{ C}}_{{{\text{copies}}/\upmu {\text{l}}}} \times { 25}{\text{.}}$$

Cell per reaction prior to droplet generation:

$${\text{C}}_{{{\text{cells}}/{\text{reaction}}}} = {\text{ C}}_{{{\text{copies}}/{\text{reaction}}}} /{ 2}$$

Copies/µl to nanograms/µl conversion (for mouse assume 3 picograms per copy or 6 picograms per cell):

$${\text{C}}_{{{\text{ng}}/\upmu {\text{l}}}} = {\text{ C}}_{{{\text{copies}}/\upmu {\text{l}}}} \times { 3 }/{ 1}000.$$

Average false positive rate:

$${\text{A}}_{{{\text{FP}}}} = {\text{ total}}\,\# \,{\text{of}}\,{\text{false}}\,{\text{positive}}\,{\text{drops}}/\# \,{\text{of}}\,{\text{reactions}}\,{\text{tested}}{.}$$

## Supplementary Information

Supplementary Figures.

Supplementary Information 1.

Supplementary Information 2.
