# Supplementary material for: Streamlined and quantitative detection of chimerism using digital PCR
Source: Sci Rep. 2022 Jun 17;12:10223. doi: 10.1038/s41598-022-14467-5 (PMC9206010; doi:10.1038/s41598-022-14467-5)
Supplement: Supplementary file 1 — Supplementary Figures. [file 41598_2022_14467_MOESM1_ESM.docx]

**SUPPLEMENTARY FIGURES**


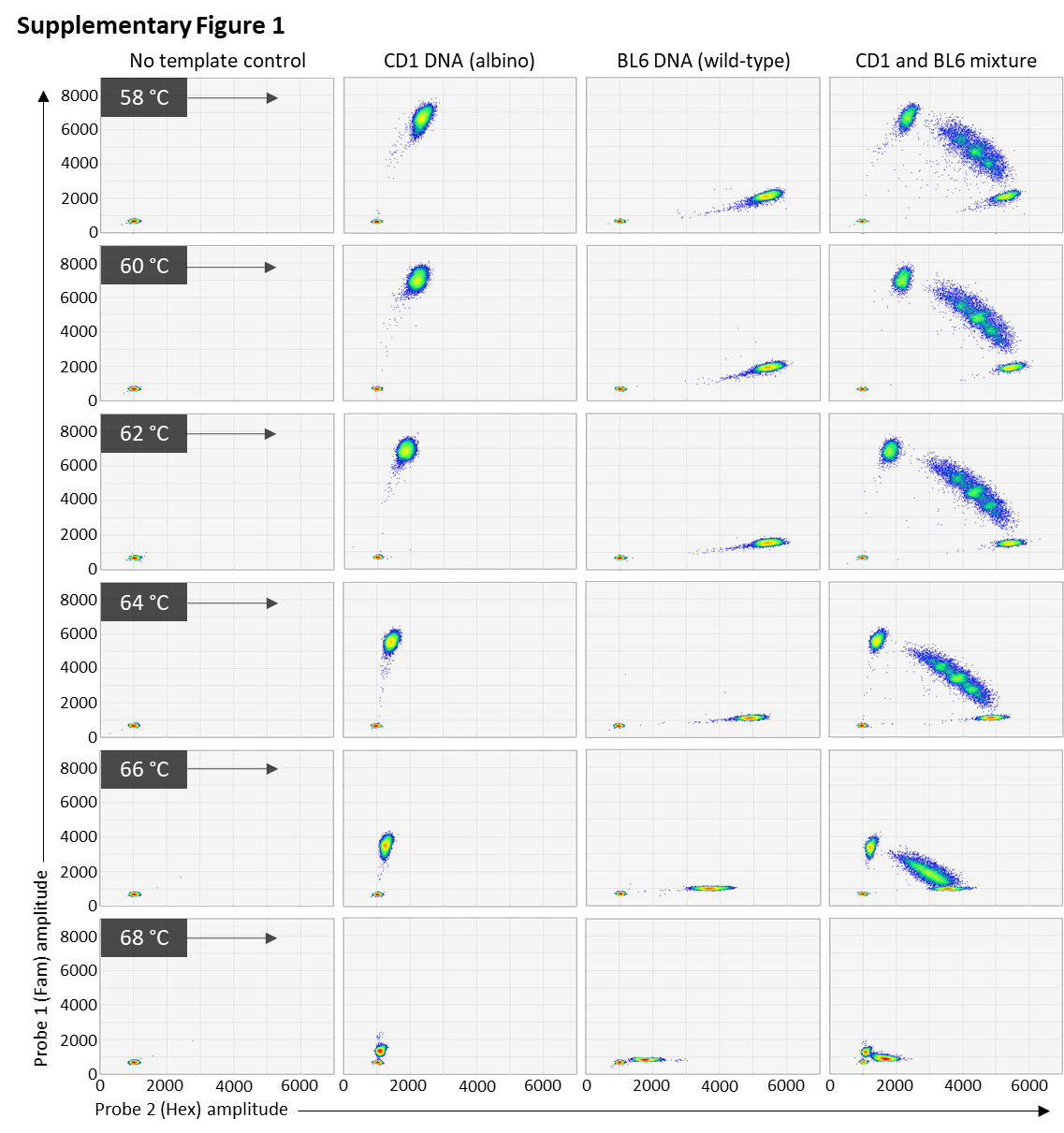


***Supplementary Figure 1 legend. Temperature optimization.***

*2D ddPCR plots of the tyrosinase SND-ddPCR assay were performed at various annealing/extension temperatures. Columns 1-4 (left to right) indicate no template control, CD1 DNA, BL6 DNA and a 1:1 mixture of CD1 and BL6 DNA respectively. Rows 1 to 5 (top to bottom) indicate the thermocycler annealing/extension step was run at 58 °C, 60 °C, 62 °C, 64 °C, 66 °C, and 68 °C respectively.*


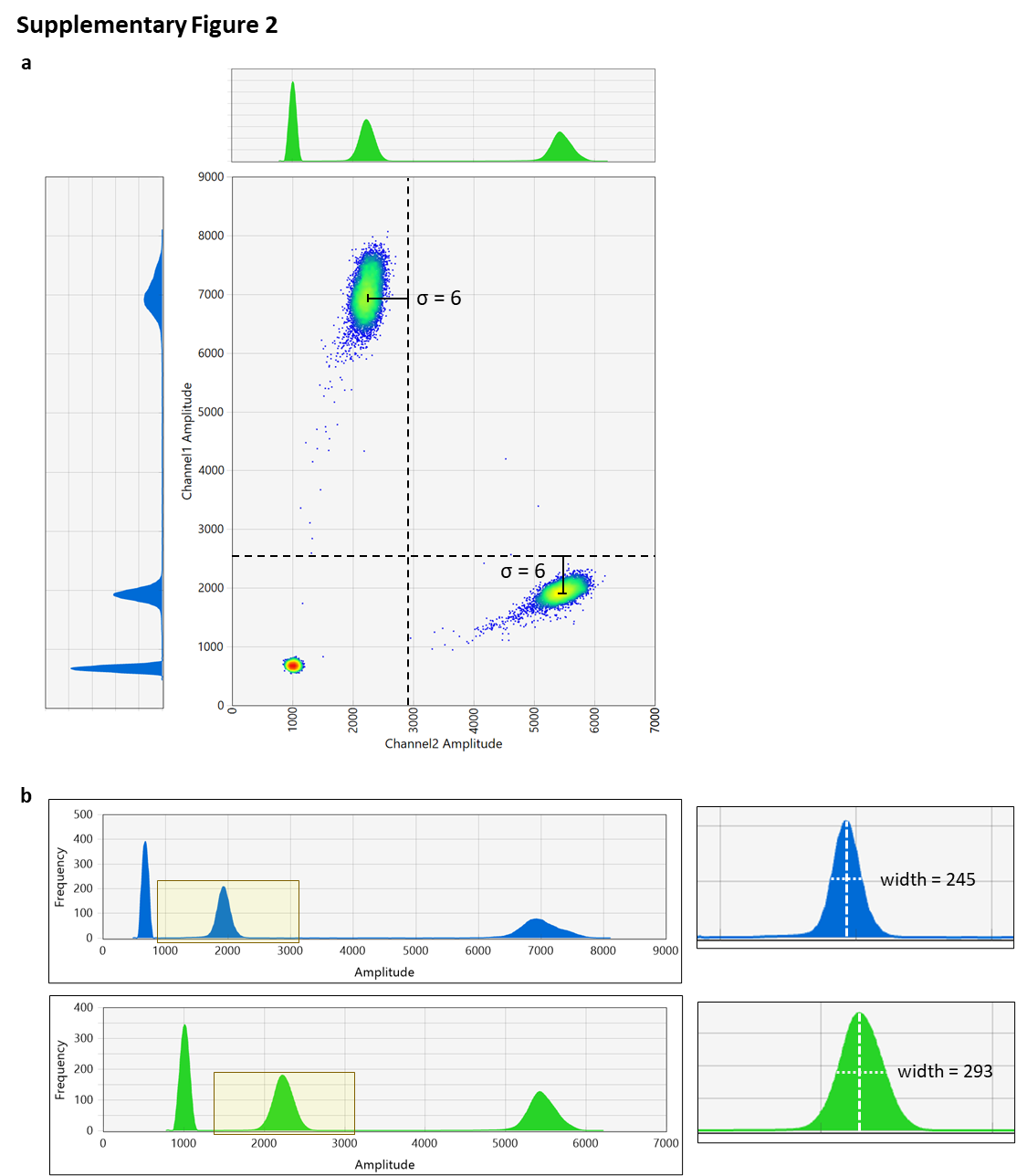


***Supplementary Figure 2 legend. Quadrant gating strategy.***

***a.*** *2D ddPCR plots of CD1 and BL6 DNA overlayed. Left and top histogram display channel 1 (y-axis, FAM) and channel 2 (x-axis, HEX) droplet-amplitude frequency, respectively.* ***b.*** *Histogram peaks highlighted with yellow boxes (left) were analyzed (right). The blue histogram (top) represents channel 1 and the green histogram (bottom) represents channel 2. White dashed lines indicate peak height; white dotted lines indicate full peak width at half max height (FWHM). FWHM is used to determine standard deviation (σ). Gates were set at 6σ from cluster centroids.*


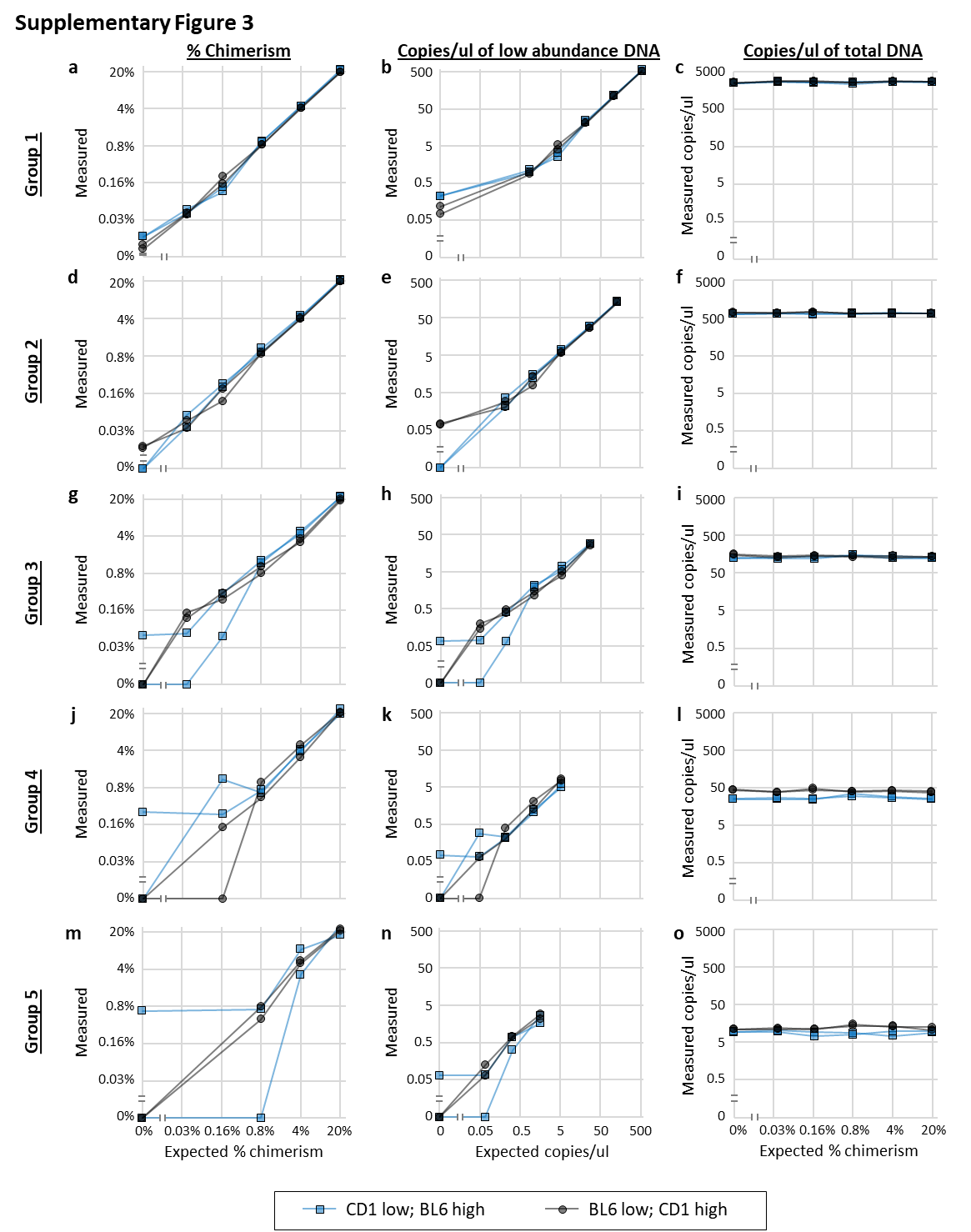


***Supplementary Figure 3 legend. CD1 and BL6 dilution matrix***

*The measured abundance of Tyr^Alb^ and Tyr^WT^ allele in mixtures of CD1 and BL6 genomic DNA; measured results compared to expected results from dilution matrix. Axes are log-scaled. Blue boxes show two separate measurements when Tyr^Alb^ is the low abundant allele (CD1 low, BL6 high). Blue lines connect a prepared dilution series within the group. Gray circles show two separate measurements when Tyr^WT^ is the low abundant allele (BL6 low, CD1 high). Gray lines connect a prepared dilution series within the group. The left column (****a, d, g, j, m****) plots the measured %chimerism of the low abundance allele in Groups 1-5, respectively, compared to the expected %chimerism. The middle column (****b, e, h, k, n****) plots the measured concentration of the low abundance allele in Groups 1-5, respectively, compared to the expected concentration. The right column (****c, f, i, l, o****) plots the measured total concentration (sum of both alleles) in Groups 1-5, respectively, for each sample within that group; they are the same in each group as expected.*


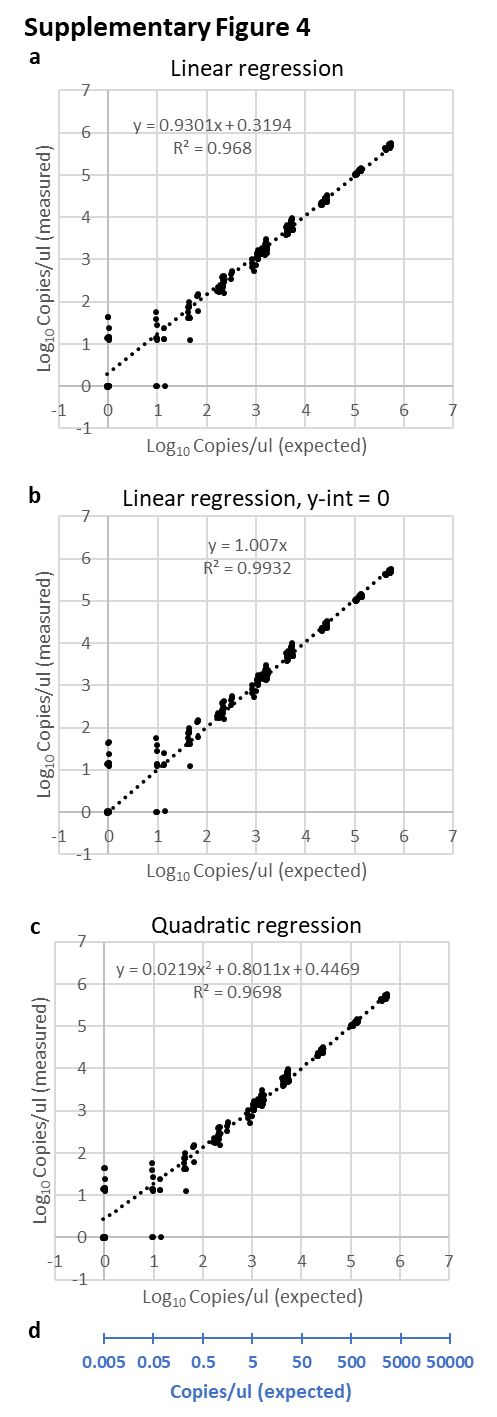


***Supplementary Figure 4 legend. Regression analysis of dilution matrix.***

***a.*** *Linear regression of log-transformed data from dilution matrix.* ***b.*** *Linear regression of log-transformed data with y-intercept set to 0.* ***c.*** *Quadratic regression of log-transformed data. For a-c, the y-axis indicates ddPCR measurement and the x-axis indicates expected concentration as determined by the dilution matrix. 0.005 copies/ul was added to each datapoint to enable log-transformation of 0 values, and 5% random error was added to aid in visualization. The points were then log-transformed and log_10_(0.005) was subtracted from each point to set the origin to 0,0. n = 108. Regression coefficients displayed on each graph.* ***d.*** *Reference markings for copies/ul.*


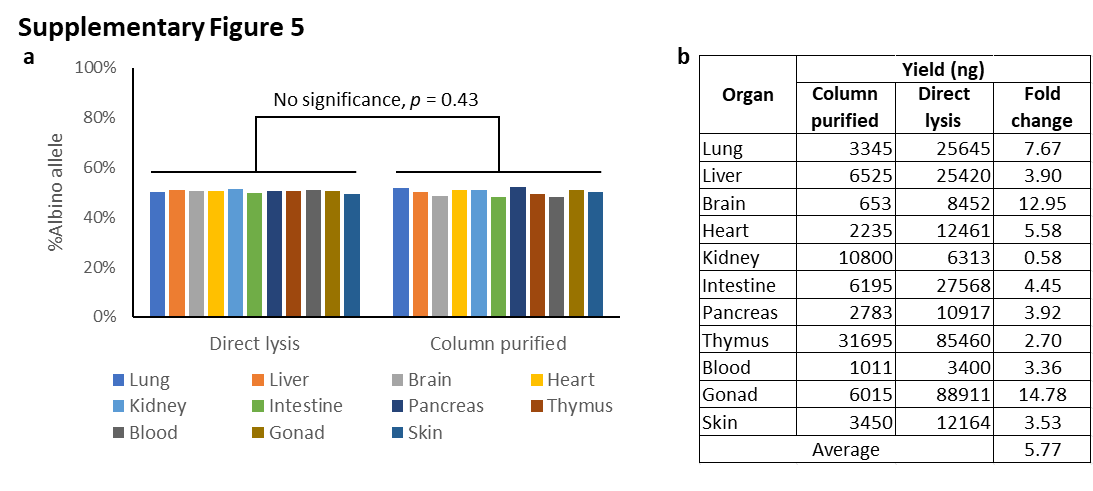


***Supplementary Figure 5 legend. Comparison of DNA extraction methods.***

***a.*** *Comparison of crude lysis buffer and column purified DNA from various organs. DNA was from CD1 x DBA/2J F1 hybrid and expected to have 50% Tyr^alb^.* ***b.*** *The total yield of DNA as determined by ddPCR.*


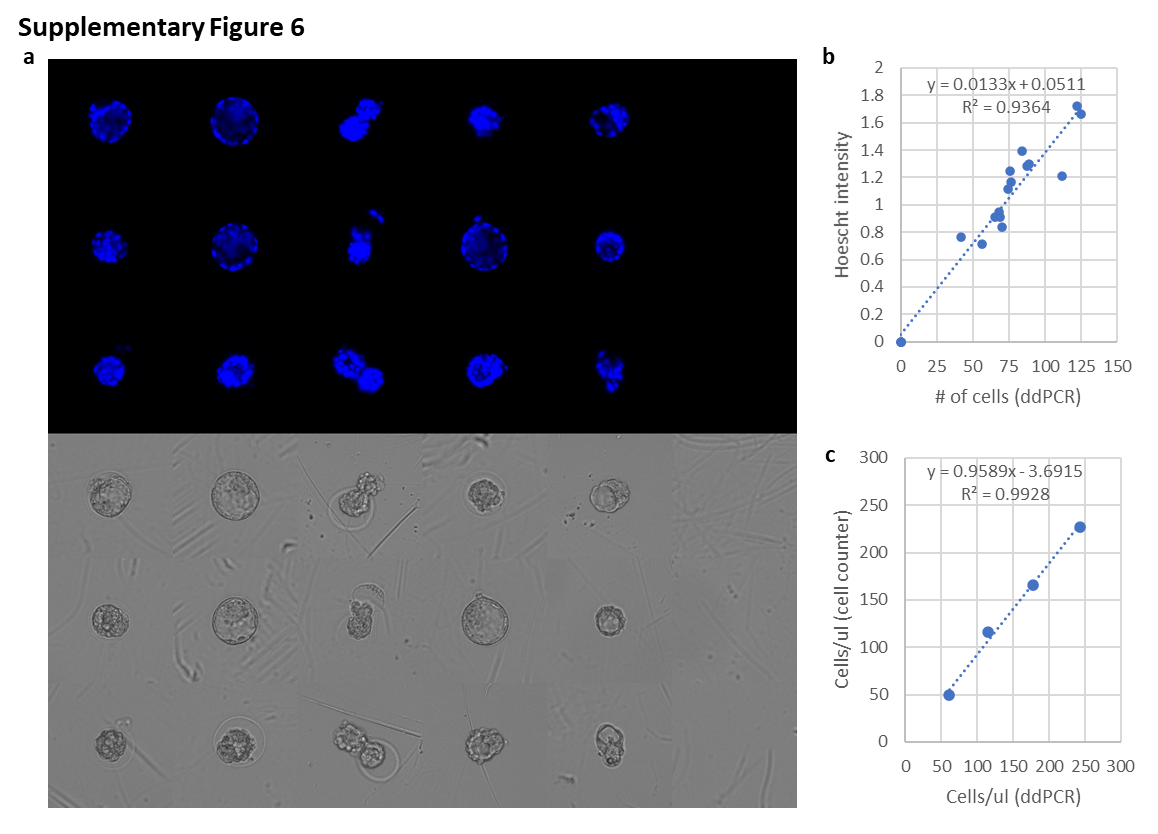


***Supplementary Figure 6 legend. ddPCR cell counting.***

***a.*** *Images of 15 embryos and 3 negative controls. The upper panel is Hoescht fluorescence (blue) and the lower is brightfield (gray).* ***b.*** *Correlation between Hoescht intensity and ddPCR cell count. The background was subtracted from each image and the total intensity was calculated.* ***c.*** *Counting mouse white blood cells at four different concentrations using ddPCR and an automated cell counter.*
